# The Influence of the Smile on the Perceived Facial Type Esthetics

**DOI:** 10.1155/2018/3562916

**Published:** 2018-07-09

**Authors:** Waeil Batwa

**Affiliations:** Orthodontic Department, Faculty of Dentistry, King Abdulaziz University, Jeddah, Saudi Arabia

## Abstract

**Objective:**

The objective of this study was to determine if the smile would influence the facial types esthetics perception for dentists, specialists, and laypeople. The null hypotheses for this study were that the smile has no effect on the perceived facial esthetics of different facial types.

**Materials and Method:**

A photograph of an attractive female face with an attractive smile was captured and manipulated using computer software, which was used to produce changes in the smile and facial type of the female face. Two sets of photographs were developed. The first set is composed of three photos showing mesofacial, dolichofacial, and brachyfacial faces; on these photos the smiles were masked intentionally. On the second set, the smile was revealed in the three face types (mesofacial, dolichofacial, and brachyfacial faces); this results in three smiling photos, where each showed a facial type with the same smile. These photos (6 photos in total) were rated by the participants; two hundred participants were recruited, 50 general dentists, 50 specialist dentists, and 100 laypeople.

**Results:**

The three groups (dentists, specialists, and laypeople) rated the mesofacial face as the highest (*p* value < 0.01) (64.48, 76.12, and 60.68, respectively), the mesofacial face was the only face that showed a significant difference between the three groups ratings (*p* value<0.01), and this significant difference disappeared when we compared the smiling photos for the mesofacial face (*p* value>0.01).

**Conclusion:**

Mesofacial face is considered to be the most attractive face in comparison to dolichofacial and brachyfacial faces. Facial type should not be looked at separately from the smile as the smile might influence the esthetics perception of the facial type.

## 1. Introduction

Facial beauty and attractiveness had been linked to social perception, where people with a more attractive face are perceived to have higher athletic, social, and leadership skills [1]. It has been found that the eyes and the mouth were the most important factors in a hierarchy of characteristics for determining facial beauty [2]. Although smile attractiveness has been linked to facial beauty in the literature [3], some authors, challenge this, claiming that dental attractiveness is not a main determent of facial beauty [4], proposing that other factors in the facial complexity play a more significant role; this includes cheeks, chin, eyes, hair, lips, nose, and skin, where all contributed equally. In a good attempt to resolve this conflict and assess the importance of dental appearance when looking at a face, an eye tracking device was used to test eyes fixation when looking at different female faces with different kinds of malocclusions; as expected, dental attractiveness was found to influence the level of visual attentions [5]. This shed the light on the complicated relationship between the smile and facial beauty. One aspect of this is the influence of face shape on the smile esthetics, specifically facial type! This relationship was investigated for some smile features such as smile line, yet no clear association was made between the smile feature and facial type [6]. It had been suggested that dentists and laypeople (but not orthodontists) preferred 2 mm gingival display with short face, but for the long face the orthodontist preferred 0 mm gingival display, while the dentists and laypeople preferred 2 mm gingival display [6]. On another attempt, it is been found that individuals with different facial types had a different perception in what constitute an attractive smile when it comes to buccal corridors [7]; candidates with mesofacial, dolichofacial, and brachyfacial faces found the mesofacial face more attractive, accepting buccal corridors range from 2 to 15%. We believe that facial types (mesofacial, dolichofacial, and brachyfacial) are thought to play a role in facial esthetics. Patients with a brachyfacial structure tend to have a shorter facial height relative to the width of the face. Patients with a dolichofacial structure tend to have a longer, narrower face. And these changes in facial dimension could influence the smile as it changes the proportion of the smile to the face.

The purpose of this study was to determine what the most attractive facial type is; dentists, specialist dentists, and laypeople assess if the smile would influence the facial types esthetics perception. The null hypothesis for this study was that the smile esthetics has no effect on the perceived facial types esthetics of different facial types.

## 2. Materials and Method

**Figure 1:**
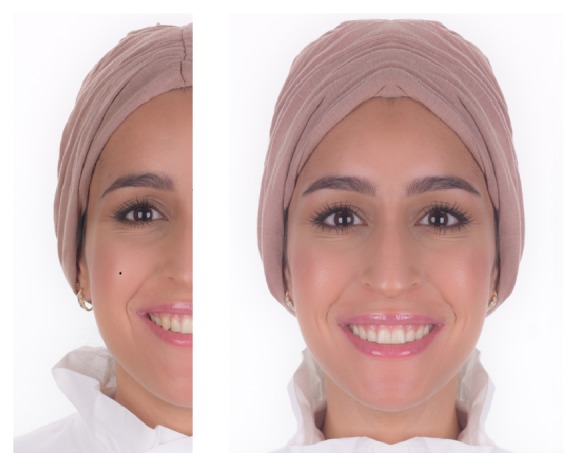
The process of creating a symmetrical face; left is selected and mirrored to develop a fully symmetrical face.

A volunteer with the following criteria was selected to obtain a frontal smiling photograph of the face: (1) an attractive mesofacial face, (2) complete dentition with well aligned anterior teeth with no rotations or crowding, (3) ideal overjet, (4) no facial asymmetry. Consent was obtained and signed by the volunteered candidate giving the permission to modify and publish the captured photos. An attractive female was selected and a photograph was captured and manipulated using Adobe Photoshop Elements software. The image was obtained by capturing a photo of the face (using Cannon EOS 40D digital camera, 10 megapixels, Tokyo, Japan), in which the camera lens was perpendicular to the long access of the face, with a standardized focal distance and depth, adjusted light condition using flash light. After loading the photograph on the computer software (Adobe Photoshop Elements 14 Editor, Adobe Systems Inc., San Francisco, California, USA), one side of the photo was selected and mirrored along the long axis of the face, to create a fully symmetrical face (Figure 1). Any skin imperfections were removed using the Photoshop. The software then was used to manipulate the face dimension by adjusting the height and width of the face, the zygomatic points (right and left), nasion, and lower point of chin (Gnathion) were used as reference points. Facial index was used as mean of determining the face type and facial index, calculated by dividing the bizygomatic width (Zyr-Zyl) on the face height (Nasion-Gnathion); a dolichofacial (long) face had a facial index less than 74.9, while a mesofacial (average) face had a range of (75-79.9) and the brachyfacial (short) face was larger than 90 [8].

**Figure 2:**
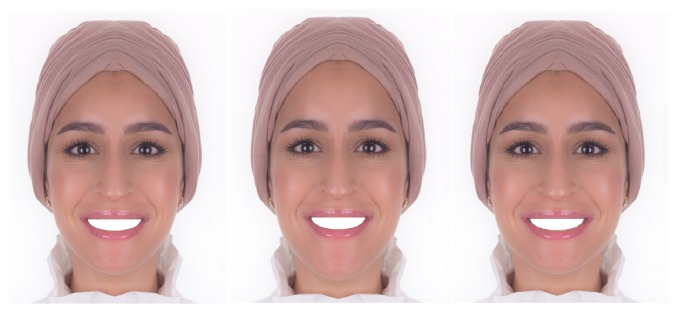
Three developed faces, from left to right, mesofacial face, dolichofacial face, and brachyfacial face. The smiles were sealed intentionally in these photos, not to distract the assessor by the teeth, while they are assessing the different face types.

**Figure 3:**
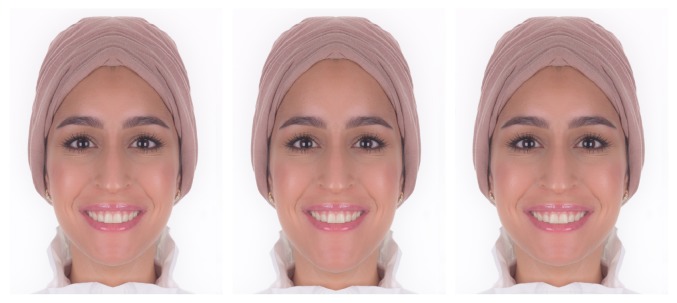
Three photos showing each facial type with a smile (meso, dolicho, and brachyfacial). The smiles added to all face types are identical.

Two sets of photographs were developed. The first set is composed of three photos showing mesofacial, brachyfacial, and dolichofacial faces; on these photos the smiles were masked intentionally (Figure 2). On the second set, three photos were developed showing each facial type with a smile (Figure 3).

*Participant Identification*. After ethical approval was obtained from Ethics and Research Committee, in the Faculty of Dentistry, laypeople aged 16 years or over were identified for possible inclusion in the study; general dentists and specialist dentists were recruited from the University Hospital. The investigator approached all participants within the target age range initially. The purpose of the study was explained. Participants were informed that they were under no obligation to participate and that they could withdraw from the study at any time. Self-completed electronic questionnaires were completed by the participants on an electronic device (iPad Pro, 10.5 inch, Apple Incorporation, California, USA) using an online survey software (Surveymonkey.com, California, USA). Each participant's name, date of birth, and gender were recorded on the questionnaire. Two hundred participants were recruited, 50 general dentists, 50 specialist dentists, and 100 laypeople. Rating of the photographs was carried out in a quiet, nonclinical environment with good lighting conditions. Participants were allowed to view each photograph for as long as they found necessary. The researcher presented each image individually on the electronic device screen (iPad Pro); the research made sure that the complete face is displayed on the screen before the participants assess it. The participants assessed the photos using visual analogue scale (Kocher, 2016 [9]); the left side of the scale was described as unattractive, while the right side was described as most attractive with a range of 0-100. To assess participant repeatability, a second set of the same photos was assessed by the participant.

All results were exported from the survey monkey online data base, to a Microsoft Excel file (Microsoft Office 365 Home, Microsoft Corporation, USA) and then copied and entered into a computer using Statistical Package for Social Science software (SPSS) (IBM SPSS Statistics for Windows, Version 21.0, Armonk, NY: IBM Corporation, USA) for analysis. All data were coded for anonymity and was computer password-protected. Participants were not informed about which features of the smile were altered, nor were they allowed to return to previous images for comparison. Researchers did not intervene. This ensured that participants' judgment was independent. The total number of assessed photos was six, three facial types (Figure 2), three facial types with smiles (Figure 3), and the same set again (six photos) for repeatability.

### 2.1. Statistical Analysis

One-way ANOVA test was used to compare the differences in images rating between the three groups and between the three photos for each set. The level of significance was adjusted at 1% level (*P* ≤ 0.01) to reduce the chance of errors with multiple testing (Type I error); any significant differences were followed by Post Hoc analysis to determine where is the difference. Analysis of data was carried on SPSS (IBM SPSS Statistics for Windows, Version 21.0, Armonk, NY: IBM Corp, United State of America)

## 3. Results

For the general dentists, the age ranged from 23 to 48 with a mean age of 27.9, while for the specialists it ranged from 30 to 64 with a mean age of 39.3; for the laypeople group, the age ranged from 16 to 60 years with a mean age of 29.8. Of the 200 recruited candidates, 81 (40.5%) were male and 119 (59.5%) were female. For the dentists group, 19 (38%) were male and 31(61%) were females; the specialist group had 20 (40%) males and 30 (60%) females, which was almost the same for the laypeople group with 42 (42%) males and 58 (58%) females. More details of sample and gender distribution can be found in Table 1. No significant difference (p > 0.01) was found when comparing male and female ratings for any of the photos (Table 2).

When comparing the three face types, the three groups (dentists, specialists, and laypeople) rated the mesofacial face as the highest (64.48, 76.12, and 60.68, respectively). Followed by dolichofacial (60.98, 66.16, and 58.23, respectively), while brachyfacial was rated the least attractive (55.4, 62.08, and 58.23). Saying this, only the specialists rated the mesofacial face significantly higher than the other two faces (*p *value < 0.01); for the other two groups the difference in ratings was not significantly different. When comparing the three groups ratings, the mesofacial face was the only face that showed a significant difference between the three groups ratings (*p* value<0.01) (Table 3). Tukey's Post Hoc analysis revealed that the significant difference is between the specialists and the laypeople ratings (*p* value <0.01), where there was no difference between the dentists and specialist nor the dentists and laypeople (Table 4).

Interestingly, when the smile was added to the facial images, the results changed (Table 5); at first, the mesofacial face was not rated significantly better than the other two faces; moreover, it was not rated significantly different between by three groups (*p* = 0.06 and* p* = 0.21, respectively). The dolichofacial face got the highest score for the specialist group 67.4 followed by the mesofacial face 66.06 and the brachyfacial face 57.34, yet the dolichofacial face was not rated significantly higher than the other two faces. Cronbach's alpha Inter-reliability test was performed; excellent level of agreement was recorded as the value was above 0.90.

## 4. Discussion

The facial beauty guidelines used by clinicians today are based on those initially described in Egyptian and Greek art and sculpture studies [10]. The assessment of facial beauty is immersed in subjectivity; nevertheless, facial proportions and facial symmetry were measured as an attempt to develop a formula to guide clinician to suggest to patient what constitute a beautiful face. We know for a fact now that what clinicians would today refer to as evidence for what constitutes ‘ideal' facial measurements is based on population averages, comes from growth studies using cephalometric radiography [11] and anthropometry [12], and has its own limitations. Perception of facial beauty and smile aesthetics varies from a person to another; it can be influenced by gender [13], age [14], and personal experiences [15]. In order to accommodate the effect of personal experiences we recruited laypeople and professionals (general dentists and specialist dentists) in this study. Moreover, we matched the gender distribution in the three recruited groups (60% female and 40% male). The gender differences in defining what constitute an attractive face and smile are evident in the literature where the disagreement between males and females regarding smile esthetics was consistent [16]; saying this, it was not the case in this study; no difference was found between male and female regarding their perception of facial type (P > 0.01). This could be due to the true lack of difference between the two genders or the small sample size which was not enough to pick the difference.

This would be comforting to orthodontists, as orthodontic diagnosis and treatment planning had changed significantly in the last few years, with a major shift toward including patient's wishes and requirements in the traditional problem oriented approach. This was driven by the increased realization of aesthetics importance, and the developed individuals own perception of their facial beauty and deformities [17]. Facial and smile attractiveness is a major objective in dentistry in general and in orthodontic in specific. Patients seek dental and orthodontic treatment for many reasons, to improve their appearance [18], function of teeth, being influenced by external factors, and because they want to improve their confidence [19]. While orthodontic treatment usually addresses the patients first two concerns, it boosts the patients confidence by treating facial deformity to it is optimum aesthetics by treating dolichofacial and brachifacial patients to the normal face via orthodontic/orthognathic treatments [20–23].

The success in treatment relies mainly on an accurate diagnosis of the problem, whether it is facial or dental. Some authors divided the esthetics components into facial esthetics, gingival esthetics, microesthetics, and macroesthetics [24], where macroethetics represents the relationships and ratios of relating multiple teeth to each other, to soft tissue, and to facial characteristics [25–28]. Microesthetics deals with the elements that make teeth actually look like teeth, such as anatomy, translucency, and shape. Whatever classification is being used, dealing with all these components is quite challenging, due to the tangled relationship between them and the increased number of smile components that could pass 14 elements [29]; one of these components is the facial type and its interaction with the smile.

Our results showed that the mesofacial type face was rated better by the three groups (64.48, 76.12, and 60.68, respectively); this finding is in agreement with Pithon et al., who found that mesofacial face is more attractive for their recruited evaluators. The same was reported by Varlik et al., who found the average face length was more attractive, and as the deviation from the norm increased the facial attractiveness gets significantly reduced [30]. Although the participants in our study rated the mesofacial face higher they were in disagreement of how much attractive it is, which is reflected by the significant difference in their rating (*P *< 0.01), where the specialist gave it the highest score which was significantly different from the laypeople score (*P *< 0.01), yet not different from the dentists score. This is simply reflecting the critical eye of the specialists [31] which scored the normal face (mesofacial) better (*P *< 0.01) than the deviated faces but again it reflects the high tolerance of laypeople who preferred the mesofacial face but to a lesser degree than the specialists.

The most interesting finding in this study would be the disappearance of the disagreement between the specialists and laypeople (*P *> .025) when the smiles were combined with the facial types, where specialists scored both smiling mesofacial faces as 66, and laypeople scored it around 65 and 63. This would mean that the smile components diluted the impact of the facial type to a level that it is not bothering the specialist no more. In other words, it means that the smile changed the perception of the facial type specially for the specialist; based on this finding we could reject the null hypothesis that the smile has no effect on the perceived facial esthetics of different facial types.

Visual analogue scale (VAS) is considered to be a simple and reliable technique. Saying this, VAS scores are subjective and variable and limitations come with it. The terms very attractive and very unattractive that represent both ends of the scale may be interpreted differently and may not convey the same feelings when used by different people. In addition, the score on the scales recorded by different people may not indicate the exact intensity of feelings [30]. Still, our participants were consistent in their ratings which was reflected in the excellent repeatability score.

The rater's gender [16, 30], age [9], education [9], self-perceived attractiveness [7], proficiency [32], and personal profile are influential in the scores of attractiveness. This needs to be considered while interpreting the results of our study; our participants were adult middle class participants and probably this limits our findings to this category

### 4.1. Clinical Relevance

Facial type should not be looked at separately from the smile, neither the smile should be assessed in separation from the face type; both should be carefully examined and assessed taking into consideration the patient's wishes [16], as some patients would exhibit some level of tolerance to some facial esthetics features, such as mild dolichofacial face type.

## 5. Conclusion

Mesofacial face is considered to be the most attractive face in comparison to dolichofacial and brachyfacial face. Facial type should not be looked at separately from the smile as the smile might influence the esthetics perception of the facial type.

## Figures and Tables

**Table 1:** Sample groups and gender distribution.

**S. No**	**Variable**	**Responses**				**Frequency (%)**	
(1)	**Sample Size (n)**	Dentists				50	
		Specialist				50	
		Lay People				100	

(2)	**Gender**	**Dentists**		**Specialists**		**Laypeople**	
		Male	Female	Male	Female	Male	Female
		19 (38)	31 (62)	20 (40)	30 (60)	42 (42)	58 (58)

**Table 2:** Gender comparison revealed no difference between male and female regarding photos assessment. Note: independent Student's t-test is performed at confidence level of 95%.

**S. No**	**Parameter**	**Male**	**Female**	**P value (F value)**
		**(n=81)**	**(n=119)**	
(1)	**Mesofacial**	64.68	66.04	0.70 (0.14)
(2)	**Brachyfacial**	57.51	55.51	0.57(0.31)
(3)	**Dolichofacial**	59.16	62.08	0.38 (0.74)

(4)	**Mesofacial Smile 1 **	65.83	62.48	0.34 (0.88)
(5)	**Dolichofacial Smile 1 **	64.19	61.92	0.47 (0.50)
(6)	**Brachyfacial Smile 1 **	59.83	56.08	0.29 (1.08)

**Table 3:** To test the difference in perception between the three groups, one-way ANOVA was performed at confidence level of 95%, and *p* value was adjusted to 0.01. ^*∗*^ p value <0.01.

**Clinical Parameters Facial Type & Smile Curvature compared between Study Groups**					
**Mean± SD**					
**S.No**	**Parameter**	**Dentists** **(n=50)**	**Specialists** **(n=50)**	**Lay people** **(n=100)**	**P value (F value)**
(1)	**Mesofacial**	64.48±24.94	76.12±18.35	60.68±25.48	**0.001** ^*∗*^ **(7.09)**

(2)	**Dolichofacial**	55.40±25.82	62.08±22.29	53.90±25	0.15(1.89)

(3)	**Brachyfacial**	60.98±23.55	66.16±20.11	58.23±24.76	0.15 (1.91)

**P value (F value)**		0.18 (1.70)	**0.002** ^*∗*^ **(6.31)**	0.15 (1.87)	

**Table 4:** Post hoc analysis revealed a significant difference between the specialists and lay people. ^*∗*^p value<0.01.

**Tukey's Post Hoc Analysis**				
**Parameter**	**Study Group**	**Dentists**	**Specialists**	**Laypeople**
**Mesofacial**	**Dentists**	-	0.04	0.62
	**Specialists**	0.04	-	0.001^*∗*^
	**Laypeople**	0.62	0.001^*∗*^	-

**Table 5:** To test the difference in perception between the three groups, one-way ANOVA was performed at confidence level of 95%, and *p* value was adjusted to 0.01. ^*∗*^ p value <0.01.

**Clinical Parameters Facial Type & Smile Curvature compared between Study Groups**					
**Mean± SD**					
**S.No**	**Parameter**	**Dentists**	**Specialists**	**Lay people**	**P value (F value)**
		**(n=50)**	**(n=50)**	**(n=100)**	
(1)	**Mesofacial Smile**	58.58±23.88	66.06±23.99	65.35±25.26	0.21 (1.53)

(2)	**Dolichofacial Smile**	56.42±23.27	67.40±19.46	63.77±21.96	0.03 (3.38)

(3)	**Brachyfacial Smile**	52.74±23.33	57.34±25.21	60.15±25.49	0.23 (1.47)

**P value (F value)**		0.45 (0.78)	0.06 (2.81)	0.30 (1.20)	

## Data Availability

Data are available in the Orthodontic Department, King Abdulaziz University, Jeddah, Saudi Arabia.
